# Emotional Accessibility Is More Important Than Sexual Accessibility in Evaluating Romantic Relationships – Especially for Women: A Conjoint Analysis

**DOI:** 10.3389/fpsyg.2018.00632

**Published:** 2018-05-14

**Authors:** T. J. Wade, Justin Mogilski

**Affiliations:** ^1^Psychology, Bucknell University, Lewisburg, PA, United States; ^2^Psychology, Oakland University, Rochester, MI, United States

**Keywords:** mate expulsion, sex, psychological methods, conjoint analysis, accessibility

## Abstract

Prior research examining mate expulsion indicates that women are more likely to expel a mate due to deficits in emotional access while men are more likely to expel a mate due to deficits in sexual access. Prior research highlights the importance of accounting for measurement limitations (e.g., the use of incremental vs. forced-choice measures) when assessing attitudes toward sexual and emotional infidelity, Sagarin et al., 2012, Wade and Brown, 2012). The present research uses conjoint analysis, a novel methodology for controlling several limitations of using continuous self-report measures in mate expulsion research. Participants (*N* = 181, 128 women) recruited from Bucknell University and several psychology recruitment listservs in the United States rated nine profiles that varied in three potential levels of emotional and sexual accessibility. Men were more likely to want to break up with a partner due to sexual accessibility deficits, whereas women were more likely to want to break up due to emotional accessibility deficits. However, regardless of sex, emotional inaccessibility was more likely to produce mate expulsion. These findings are consistent with prior theory and highlight the need to disentangle emotional accessibility into its constituent in-pair benefits. This research also illustrates the utility of conjoint analysis as a statistical tool for studying how humans resolve trade-offs among competing outcomes during romantic decision-making.

## Introduction

Men and women face challenges when selecting, attracting, and retaining mates. Prior research documents robust sex differences in men’s and women’s typical mate preferences (see Schmitt, 2015, for a review), mate retention behaviors (e.g., Lopes et al., 2017), and reactions to real or hypothetical partner loss (e.g., Kuhle, 2011). Men tend to report greater distress imagining a partner’s sexual contact with another man whereas women report greater distress from a partner’s emotional or financial investment in another woman (see Sagarin et al., 2012, for a review). Likewise, recent work suggests that romantic conflict reconciliation (Wade et al., 2017) and romantic relationship dissolution (i.e., mate expulsion; Wade and Brown, 2012) follow similar patterns. For example, Wade et al. (2017) asked men and women to rate the effectiveness of several reconciliation behaviors in resolving romantic conflict. Men reported that a partner’s sexual accessibility (e.g., giving sex/sexual favors) would be more effective than did women, whereas women reported that “spending time together, crying, and apologizing” were more effective. This research suggests that sex differentiation in romantic cognition and its resultant behavior extends to decisions about whether to expel a current mate.

These sex differences have been shaped by the adaptive problems that men and women typically encounter when deciding to maintain or dissolve a romantic pair-bond (see Edlund and Sagarin, 2017, for a review). Insofar as sexual access is paramount to male mate selection and partner commitment is principle during women’s mate selection, one might expect a partner’s sexual or emotional accessibility to influence mate expulsion. A partner’s willingness to have sex maybe most important for men’s relationship termination decisions. A female partner’s withdrawal of sex compromises her reproductive potential and may diminish her partner’s paternity certainty. Thus, one would expect men to be most distressed by a partner’s withdrawal of sexual access. Conversely, a partner’s willingness to invest time and resources into the pair-bond (i.e., emotional accessibility) may be most important for women’s relationship termination decisions. That is, women may become more distressed by a male partner’s emotional inaccessibility or withdrawal of valuable in-pair investment (e.g., time, money, or interpersonal support). This dearth of emotional support or intimacy may indicate that he no longer loves his partner or is unwilling to sufficiently devote effort toward her (Buss, 1988; Wade et al., 2009). Certainly, third parties who offer this investment are perceived as more likely to be successful in facilitating partner defection (i.e., mate poaching; Schmitt and Buss, 2001; Mogilski and Wade, 2013).

Few studies have examined sex differences in relationship termination due to partner inaccessibility. Wade and Brown (2012) examined how incremental deficits in sexual access and emotional access affect relationship termination decisions using an evolutionary theory perspective. Consistent with *a priori* predictions, they found that a lack of emotional access led to mate expulsion for women and a lack of sexual access led to mate expulsion for men. From this, Wade and Brown (2012) concluded that men and women differ in the importance they place on a partner’s sexual and emotional access for mate expulsion decisions. Nevertheless, it is possible that these differences were due to the manner by which participants were asked to assess emotional and sexual accessibility deficits. Prior work suggests that asking participants to assess the salience of a partner’s infidelity using Likert-type or forced-choice measures limit the conclusions that researchers may draw about sex differences in reaction to sexual versus emotional stimuli in evolutionary psychological studies (DeSteno et al., 2002). Specifically, these methods prevent researchers from drawing conclusions about how important a partner’s sexual versus emotional accessibility is when assessed alongside the other. For example, in forced-choice designs, participants are limited to expressing their reaction to one type of infidelity versus the other while assuming that the other type is non-existent (e.g., “How upset would you be if your partner was emotionally accessible but sexually inaccessible?”). In naturalistic environments, this situation is unlikely to occur, and the extent to which a partner’s extra-dyadic behaviors entail emotional or sexual involvement may vary. Similarly, asking participants to react using continuous measures (e.g., “On a scale from 1 (not at all) to 7 (extremely), how upset would you be if you caught your partner having sex with [or falling in love with] someone else?”) allows participants to assess sexual and emotional accessibility independently. This prevents researchers from drawing conclusions about how important each type of access would be if assessed relative to the other.

### Conjoint Analysis

To address these limitations, we implemented conjoint analysis to examine how men and women prioritize a partner’s sexual versus emotional accessibility when asked to assess them within partner profiles. Conjoint analysis is a popular multivariate analysis within marketing research (Gustafsson et al., 2007; Lohrke et al., 2010) and is a novel analytic tool within human mating research (e.g., Mogilski et al., 2014; Mogilski and Welling, 2017). This technique is used to study how individuals prioritize constituent features during holistic evaluation of multi-attribute romantic partners. Participants are asked to rank several versions of a partner, wherein each version consists of differing combinations of each feature under investigation. From these rankings, conjoint analysis provides the researcher with estimates of the relative contribution of each attribute to participants’ overall evaluations of the product.

Adopting a conjoint approach to studying romantic decision-making is advantageous insofar as human mating resembles a “mating market” (Noë, 2001) whereby individuals present versions of themselves to potential partners and attempt to secure the best quality mate given their own circumstances and market value. Research into human mate choice and partner preferences thereby nicely parallels marketing research, except that the “products” are potential mates. In the first study to use this technique to investigate men’s and women’s assessments of potential long- and short-term romantic partners, Mogilski et al. (2014) used CA to assess the relative importance of a partner’s history of sexual fidelity relative to four other mate attributes: physical attractiveness, financial stability, emotional relationship investment, and partner similarity. Using a fractional-factorial design (Hair et al., 1995), they generated an orthogonal array of 19 hypothetical partner profiles, each composed of a different combination of mate attributes. Attributes were assigned three potential levels reflecting undesirable, moderately desirable, and highly desirable amounts. For example, an individual might be described as “high in physical attractiveness, low in financial stability, high in sexual fidelity, low in emotional investment, and medium in partner similarity.” Participants were then instructed to rank these 19 profiles by their preference to start a long- and short-term relationship with each individual described. Using CA, they found that both men and women prioritized a potential long-term partner’s history of sexual fidelity over each other attribute and prioritized a potential short-term partner’s history of sexual fidelity, physical attractiveness, and financial stability over a partner’s emotional investment and similarity. By forcing participants to evaluate potential partners holistically, CA differs from traditional procedures in that it measures the relative heuristic value of individual attributes within the context of complex, multivariate personality profiles. In this sense, CA is a simple yet elegant technique for studying how individuals prioritize certain partner characteristics at the cost of losing others.

CA allows researchers to draw conclusions about how traits are prioritized by virtue of how the data are collected from participants. Studies measuring mate preferences typically obtain both independent and dependent variables from participants and then use these variables to estimate a predictive model. This is referred to as a “compositional” model (Tabachnick and Fidell, 2013). By contrast, CA uses a “decompositional” model, whereby researchers specify levels for each independent variable beforehand and present participants with profiles containing different combinations of these levels. Participants provide rankings of these profiles as dependent variables and the researchers create a predictive model by using CA to “decompose” these ratings into estimates of how important each attribute is to a participant’s ranking decisions.

This decompositional approach has several inherent benefits for studying romantic decision-making. First, it can be difficult for individuals to verbalize their internal preferences (Wilson and Dunn, 1986), and retrospective or imagined scenarios can cause response revisionism based on social desirability, faulty memory, or inability to articulate decision-making processes (Shepherd and Zacharakis, 1997). CA avoids these problems by presenting profiles that participants rank in real-time (Lohrke et al., 2010). Second, rather than rating attributes independently, participants consider the importance of attributes relative to each other attribute. In this way, participants’ hypothetical romantic evaluations reflect their preferences for an entire individual as opposed to isolated features of an individual. Finally, participants are presented with a limited number of potential partners to rank. When participants report ideal mate preferences, they may mentally sample from an unlimited pool of potential mates. Lenton et al. (2009) found that people seem to adopt a less time-consuming, non-compensatory strategy in which they use fewer, easily assessed cues, such as physical attractiveness (Kurzban and Weeden, 2005), and make fewer trade-offs when selecting a mate from a larger sample of options. Lenton and Stewart (2008) also found that participants were more likely to use a non-compensatory strategy when choosing from a large set of 64 web-dating profiles than from a small set of four profiles. This suggests that participants may use more or fewer mate attributes to select a mate when choosing from a limited versus unlimited pool of potential mates, respectively. Relatedly, participants may form preexisting assumptions about features not under investigation when the number and quality of attributes being investigated are not restricted. CA restricts the number of attributes evaluated by participants by using predefined levels for each attribute. Arguably, this presents participants with a more realistic pool from which to select hypothetical mates.

### Current Study

In the present research we applied conjoint analysis to studying mate expulsion decisions by having male and female participants rate a collection of nine profiles, each depicting differing amounts of sexual and emotional partner accessibility. In line with previous research, women were expected to rank large deficits in emotional access as more likely to lead to mate expulsion while men were expected to rank large deficits in sexual access as more likely to lead to mate expulsion.

## Materials and Methods

### Participants

Participants (*n* = 181, 128 women, 53 men; age: *M* = 21.17, *SD* = 6.24, range = 17–56) were recruited from undergraduate psychology courses at Bucknell University, and from online listservs. Reported racial composition was 89.5% White, 3.3% Black, 3.3% Asian, and 3.3% Hispanic. Participants were also asked to report whether they were current in a romantic relationship (39.2%), single (50.8%) or unsure about their relationship status (9.9%), if they identified as heterosexual (93.9%), homosexual (2.8%) or other (3.3%), and whether they were currently using any form of hormone-based contraception (men: no = 100%; women: no = 52.3%, yes = 46.9%). Undergraduate students were compensated with course credit for participation. This research was reviewed by the local IRB.

### Procedure

All experimental materials were presented using Qualtrics, an online browser-based survey software program. After completing the informed consent statement, participants were asked to think of a committed romantic relationship that they have had in the past, that they have now, or that they would like to have and imagine that there is a problem in that relationship. They were then presented with a collection of nine profiles, each depicting differing amounts of their partner’s sexual and emotional access. Sexual and emotional access were defined for participants as follows:

**Sexual accessibility** refers to how often your partner is interested in having sex or engaging in any sexually gratifying activities with you.

**Emotional accessibility** refers to how often and to what degree your partner is emotionally available and open to you.

An orthogonal array of nine profiles was generated using IBM SPSS 21 (see **Table 1**). Each profile was presented as a unique combination of emotional and sexual access, representing “high,” “medium,” and “low” access. For example, participants might have been shown partners who were “high in sexual access, but low in emotional access,” “low in sexual access, but medium in emotional access,” etc. Participants then presented the text below and asked to rank these profiles relative to one another by how likely would break up with their partner if this were how sexually/emotionally accessible they were:

**Table 1:** Orthogonal array of partner profiles and their respective attribute variations.

Profile variation	Sexual accessibility	Emotional accessibility
1	Low	Low
2	Low	High
3	High	Low
4	Medium	High
5	Medium	Medium
6	Medium	Low
7	High	Medium
8	Low	Medium
9	High	High

*Each scenario is listed on the left side of your browser. The boxed area to the right of the scenarios is where you will sort and rank each scenario. To do this, you simply have to left-click and drag each scenario into the box. You can also left-click and drag each scenario around within the box in case you wish to reorganize them. The scenario for which you would* ***MOST*** *likely break up with your partner should be rated* ***highest*** *whereas the scenario for which you would* ***LEAST*** *likely to break up with your partner should be rated* ***lowest***. *Please rank the following for* ***how likely you would break up with your partner if this were how sexually/emotionally accessible he/she was***. *Please take your time and be thorough and honest in your rankings. Once you have organized all of the scenarios, please review your rankings to make sure they are accurate.*

## Results

Conjoint analysis was performed (see Hair et al., 1995) to assess the relative importance of sexual and emotional access on participants’ profile ranking decisions. Mean importance values were calculated, which characterize the relative importance of each attribute (i.e., emotional and sexual accessibility) by comparing the range in utility estimates of each level within each attribute (i.e., high, medium, and low). Utility estimates are analogous to regression coefficients and provide a measure of preference for each attribute level. As the range among these estimates increases, this indicates that a change between high, medium, and low for an attribute produces a proportionally greater shift in participants’ evaluations of each profile. Therefore, importance values provide a percentage estimate of the relative utility of changing one trait relative to another. Importance values for all attributes collectively sum to 100.

A 2(sex) × 2(type of access) repeated measures ANOVA was performed to examine whether there were sex differences in importance values for sexual and emotional access. There was a significant interaction for sex and type of access, *F*(1,179) = 10.22, *p* = 0.002. Two *post hoc* independent samples *t*-tests with Bonferroni corrections revealed that men’s importance values were significantly higher than women’s importance values for sexual accessibility, *t*(179) = 3.20, *p* = 0.002, whereas women’s importance values were higher than men’s importance values for emotional accessibility, *t*(179) = -3.20, *p* = 0.002, see **Table 2**. Additionally, a significant main effect for type of access occurred, *F*(1,179) = 10.42, *p* = 0.001. Ignoring sex of participant, importance values for emotional access were higher than importance values for sexual access, *F*(1,179) = 10.422, *p* = 0.001 (emotional access: *M* = 58.05, *SD* = 22.20; sexual access: *M* = 41.95, *SD* = 22.20).

**Table 2:** Mean perceived importance of accessibility type across sex.

Accessibility value	Sex	Mean
Sexual	Male	49.95 (22.46)^a^
	Female	38.64 (21.32)^a^
Emotional	Male	50.05 (22.46)^b^
	Female	61.36 (21.32)^b^

*Higher numbers indicate greater importance, standard deviations are in parentheses. Superscripts denote significant comparisons, *p* < 0.05.*

## Discussion

In deciding whether to terminate a romantic relationship, women prioritized a partner’s emotional accessibility relative to his sexual accessibility whereas men prioritized a mate’s sexual accessibility relative to her emotional accessibility. These results are consistent with Sexual Strategies Theory (Buss and Schmitt, 1993; Buss, 1997). Since men have a lower parental investment need than women (Trivers, 1972), men may be less likely to maintain relationships with women who have low sex drive, are prudish, or are otherwise disinterested in sex with him—all qualities that signal lack of sexual accessibility. By comparison, women may have a lower threshold for a partner’s emotional inaccessibility insofar as partner’s in-pair commitment enhanced offspring investment (Trivers, 1972; Buss and Schmitt, 1993; Buss, 1997), particularly for long-term mating.

Prior research on reactions to infidelity shows that men and women’s reactions to a partner’s commission of infidelity are also related to the emotional access and sexual access of their partners. Shackelford and Buss (1997) and Wiederman and Kendall (1999) report that men are more upset by a partner’s sexually infidelity while women are more upset by a partner’s emotional infidelity. Men’s greater upset for a partner’s commission of sexual infidelity occurs because his sexual access (and paternity certainty) is being curtailed by another man’s efforts. Women’s greater upset for a partner’s commission of emotional infidelity occurs because their access to a partner’s time and support is being curtailed by another woman. Relatedly, Shackelford et al. (2002) report that men are also less likely to forgive a partner for committing sexual infidelity while women are less likely to forgive a partner for committing emotional infidelity. A commission of sexual infidelity could restrict or curtail a man’s sexual access to his partner (i.e., she may be less interested or available for sex with him), and a partner’s commission of emotional infidelity could indicate that a women’s partner is less interested in or available for in-pair support.

Also, sexual access plays an important role in sexual conflict for men and in relationship satisfaction (Betzig, 1989; Buss, 1989a; Wade and Brown, 2012). Men place a premium on women’s ability to reproduce (Betzig, 1989; Buckle et al., 1996). Consistent with this, Shackelford and Buss (1997) report that competition among men for sexual access to reproductively valuable women is more intense than competition among women for reproductively valuable men. Thus, perhaps not surprisingly, Sprecher and Cate (2004) report that men are less satisfied overall when their wives are sexually withholding. Similarly, Buss (1989a) reports that men report the greatest anger and upset over women who accepted resources from them but failed to provide sexual access in return. Consistent with this, Felmlee et al. (1990) report that sexual intimacy is a positive predictor of relationship stability. By contrast, women desire men who are willing to invest their resources and who are willing to commit in the long-term context (Buss, 1989b, 1997, 2006; Buss and Schmitt, 1993). Since women typically desire a larger parental investment from their male partners (Trivers, 1972), women also desire a long-term commitment from their male partners (Buss, 1989b), and interpersonal investment and love are facilitated by emotional intimacy (Aron and Fraley, 1999). Therefore, a male partner’s willingness to share his feelings/show his love for his partner or is committed (emotional accessibility) is very important for women.

Interestingly, we also found that emotional accessibility was overall more important than sexual accessibility when ignoring participant sex. These results are consistent with prior work (Wade et al., 2017) showing that men and women rate emotional commitment tactics as most effective for achieving reconciliation after romantic conflict. Likewise, Wade et al. (2009) report that men and women, overall, rate love acts that show emotional commitment as most effective for expressing love within a long-term pair-bond. Emotional accessibility may be more important overall for long-term relationships insofar as partner commitment entails benefits for both men (e.g., greater paternity certainty) and women (e.g., greater commitment certainty).

### Limitations and Future Directions

We did not include holdout profiles in the present study to test for reliability of participants’ rankings of each profile. Hold-out profiles are produced with the orthogonal array and ranked alongside other profiles, but are not used to generate the predictive model. Instead, the predictive model is generated from participants’ rankings of non-holdout profiles and then used to predict how the hold-out profiles should have been ranked by each participant. This provides a correlation coefficient (tau) showing how accurately the model predicts participants’ rankings of the hold-out profiles relative to the others. Because participants rated all possible profile permutations, all holdout profiles would have been copies of existing profiles. Though we did not use holdout profiles in this study, prior studies using similar methodologies have shown strong tau coefficients (Mogilski et al., 2014; Mogilski and Welling, 2017). Nevertheless, future research using this technique should be careful to include holdout profiles in the manner described in prior studies. Additionally, the profiles in the current research were hypothetical. Additional research including actual profiles may add even greater strength to the findings of the current research.

Future work should distinguish facets of emotional accessibility. Though traditional evolutionary models emphasize women’s relatively greater desire for partner investment (e.g., Buss and Schmitt, 1993), this investment may be allocated by a mate in diverse ways. For example, a partner could generate protection (Bleske-Rechek and Buss, 2000, 2001; Lewis et al., 2011), child care (Bouchard and Lee, 2000), social status (Felmlee, 2001), or emotional support (Schutte et al., 2001). Certainly, sexual selection has crafted long-term romantic decision-making to maximize a partner’s investment, but the manner by which this investment is provisioned may alter how someone responds to a partner’s emotional accessibility. For example, if a woman is dependent on her partner because the partner’s absence exposes them to risky or unstable conditions (e.g., environments with high male-male competition and wealth; see Little et al., 2013), she may be more willing to sacrifice her partner’s extradyadic sexual accessibility insofar as he continues to provides physical protection, the social or material capital to support her and her offspring, or enough intimacy to feel in-pair security. Parsing partner accessibility into its constituent benefits (e.g., protection, child care, social influence, genetic health) may clarify which features of emotional investment are prioritized within a given mating market, and which aspects of a romantic relationship women are less willing to sacrifice. Likewise, not all sexual behavior is equally likely to result in cuckoldry. It is possible that consistent inaccessibility of certain sexual behaviors (e.g., sexual intercourse, oral sex, kissing) is relatively more distressing than others.

## Conclusion

These findings support and add robustness to previous findings (Wade and Brown, 2012) and confirm that sexual access is prioritized for men’s mate expulsion decisions and emotional access is prioritized for women’s mate expulsion decisions. Furthermore, this research demonstrates the utility of conjoint analysis for studying mate expulsion decisions and human mating more generally. Compared to prior methods (e.g., Likert-type and forced-choice measures) conjoint analysis assesses how men and women heuristically prioritize a partner’s sexual and emotional access when they weigh and compare each within a single individual. This allows researchers to determine which factors from among several are relatively more influential for mate expulsion decisions.

## Notes

A version of this manuscript was presented at the 7th Northeastern Evolutionary Psychology Society Conference, May 30–June 2, 2013, Lebanon Valley College.

## Ethics Statement

This paper conforms to the Ethical Standards of the American Psychological Association. It was approved by the Institutional Review Board at Bucknell University. Participants signed an informed consent statement prior to taking part in the research.

## Author Contributions

Each author contributed writing and data analysis to this manuscript/research. JM contributed data collection also.

## Conflict of Interest Statement

The authors declare that the research was conducted in the absence of any commercial or financial relationships that could be construed as a potential conflict of interest.
